# Common Perceived Barriers and Facilitators for Reducing Sedentary Behaviour among Office Workers

**DOI:** 10.3390/ijerph15040792

**Published:** 2018-04-18

**Authors:** Carla F. J. Nooijen, Lena V. Kallings, Victoria Blom, Örjan Ekblom, Yvonne Forsell, Maria M. Ekblom

**Affiliations:** 1 The Department of Public Health Sciences, Karolinska Institutet, 11365 Stockholm, Sweden; yvonne.forsell@ki.se; 2 The Swedish School of Sport and Health Sciences (GIH), 11486 Stockholm, Sweden; lena.kallings@gih.se (L.V.K.); victoria.blom@gih.se (V.B.); orjan.ekblom@gih.se (Ö.E.); maria.ekblom@gih.se (M.M.E.); 3 The Department of Neuroscience, Karolinska Institutet, 17177 Stockholm, Sweden; 4 Centre for Epidemiology and Community Medicine, Stockholm Health Care District, 11365 Stockholm, Sweden

**Keywords:** sedentary behaviour, workplace, office workers, barriers, facilitators

## Abstract

Qualitative studies identified barriers and facilitators associated with work-related sedentary behaviour. The objective of this study was to determine common perceived barriers and facilitators among office workers, assess subgroup differences, and describe sedentary behaviour. From two Swedish companies, 547 office workers (41 years (IQR = 35–48), 65% women, 66% highly educated) completed questionnaires on perceived barriers and facilitators, for which subgroup differences in age, gender, education, and workplace sedentary behaviour were assessed. Sedentary behaviour was measured using inclinometers (*n* = 311). The most frequently reported barrier was sitting is a habit (67%), which was reported more among women than men (Χ^2^ = 5.14, *p* = 0.03) and more among highly sedentary office workers (Χ^2^ = 9.26, *p* < 0.01). The two other most reported barriers were that standing is uncomfortable (29%) and standing is tiring (24%). Facilitators with the most support were the introduction of either standing- or walking-meetings (respectively 33% and 29%) and more possibilities or reminders for breaks (31%). The proportion spent sedentary was 64% at the workplace, 61% on working days, and 57% on non-working days. This study provides a detailed understanding of office workers’ ideas about sitting and means to reduce sitting. We advise to include the supported facilitators and individualized support in interventions to work towards more effective strategies to reduce sedentary behaviour.

## 1. Introduction

A substantial proportion of life is spent at work, and ever more people have office jobs in which sitting is the default [1]. Epidemiological studies report that office workers spend at least two-thirds of their workday sitting [2,3]. Occupational-related sedentary behaviour is a major public health problem because there is accumulating evidence that sedentary behaviour is related to increased risk of cardiometabolic health and premature mortality [1,4].

Although sit-stand desks are not the default in many countries around the world, in Sweden most workplaces are provided with these desks. However, having a sit-stand desk does not automatically mean it is being used to shift from sitting to standing. In a study among different companies in Sweden, 60% of men and women who had recently been provided with sit-stand desks reported using them only once a month or less [5]. Another study among call-centre companies found that sit-stand desks were associated with a decrease of only 5% of the overall sitting time [6]. A Cochrane review confirmed that there is very-low- to low-quality evidence that sit-stand desks reduce workplace sitting, with no evidence on long-term effects [7].

Recently, qualitative studies identified barriers and facilitators associated to office work-related sedentary behaviour [8,9,10]. Barriers that have been identified are social norms around appropriate workplace behaviour, workload pressures, and the physical work environment. Identified facilitators having a definite purpose to sit less at work included standing meetings, relief of physical or mental symptoms of prolonged sitting, and a cultural shift in workplace norms. Another recurrent theme in the qualitative studies was lack of motivation [8,9,10]. However, since all available evidence is based on qualitative data in small samples, it is impossible to determine the universality of these barriers and facilitators. Moreover, it remains to be elucidated whether barriers and facilitators differ according to demographic characteristics and sedentary behaviour.

Although some interventions have been found effective in decreasing workplace sedentary behaviour, studies show inconsistent results and it therefore remains unknown which types of interventions and delivery modes should be advised [7]. Further study on workplace sedentary behaviour is thus warranted, and the present study aimed to contribute by providing a more in-depth understanding of the ideas that workers have about sitting and means to reduce sitting. Identifying the most common barriers and facilitators and which of these apply to certain office workers will help when developing feasible, acceptable, and potentially more effective approaches to reduce workplace sedentary behaviour.

The objective of this study was to determine the most common perceived barriers and facilitators among office workers. Furthermore, we assessed whether these barriers and facilitators were different based on age, gender, education, and objectively measured workplace sedentary behaviour. Additionally, objectively measured behaviours in- and outside of work were described. Our study expands on the currently existing literature with a large quantitative study on perceived barriers and facilitators. Furthermore, this study is the first to describe the severity of sedentary behaviour in Sweden both during and outside office hours by using state-of-the-art technology among a large group of office workers [11]. The gained knowledge is of importance for public health professionals and interventionists, and could help companies making decisions on how to decrease health risks of office workers.

## 2. Materials and Methods

### 2.1. Study Design

This project is part of the “Physical activity and healthy brain functions project” and involves two Swedish private companies which differ by size and industry. Neither of the companies had implemented a formal program to reduce sedentary behaviour.

### 2.2. Participants

All employees at the companies were invited to participate via email (*n* = 2024). Most potential participants at each company had predominately desk-based jobs and were provided with a simple electronic sit-stand desk. Participants were asked to fill out a web-based questionnaire during working hours and they were further asked to participate in an additional set of measurements, including wearing an inclinometer. Ethical approval was granted by The Stockholm Regional Ethical Review Board (2016/1840-32) and all participants provided written informed consent.

### 2.3. Measures

#### 2.3.1. Perceived Barriers and Facilitators

Based on previous qualitative research [8,9,10], we developed the first available questionnaire to assess perceived barriers and facilitators for workplace sedentary behaviour (Table S1). From these previous studies, common identified barriers and facilitators were extracted and summed up. A total of 13 barriers were identified and participants were requested to answer whether each of these applied to them, e.g., sitting is a habit, I exercise enough, standing disturbs others, or standing reduces work performance. A total of 11 facilitators were identified, and participants were asked to indicate options they would like from their employer to facilitate change, such as redesigning the workplace, use of app or activity watch, and standing- or walking-meetings. For both questions, participants were instructed to tick the box for those barriers or facilitators which applied to them, with the possibility to tick multiple boxes. For each of the barriers and facilitators, we calculated the percentage of participants agreeing by dividing the number of participants who ticked the box by the total number of participants who completed the questionnaire.

#### 2.3.2. Sitting, Standing, and Walking

ActivPAL3 activity monitors (PAL Technologies limited, Glasgow, UK) were used to measure sedentary behaviour. Devices were waterproofed and secured to the frontal aspect of the midthigh using a 10 × 10 cm adhesive hypoallergic thin plastic film (Tegaderm Roll, 3M). Participants were asked to wear the device for 7 consecutive days (24 h/day) and to record sleep and waking times, working hours, and any device removals in a diary. The devices were initialized and processed using the activPAL software version 7.2.32 (PAL Technologies limited, Glasgow, UK), using references on waking and working hours from participants’ diaries. Additional data processing was conducted using the HSC analysis program (developed by Dr. Philippa Dall and Professor Malcolm Granat, School of Health and Life Sciences, Glasgow Caledonian University). Quality controls were conducted before and after processing. Recorded time was coded as wear time, non-wear time, or working time. Non-wear time and sleep were excluded from the analyses. Working time included lunch but excluded working from home during evenings. All first days were excluded because monitors were mounted during a working day and therefore did not represent a full day. All other days were considered valid when ≥10 h of worn waking hours, <95% of time spent in any one behaviour (sedentary, standing, walking), and ≥500 steps [11]. Additionally, working days were considered valid when worn for ≥80% of the time at work and ≥5 h of worn working hours. A minimum of data from any 4 days, with at least 2 working days and 2 non-working days, were required to be included in the analytical sample. Time spent sitting, standing, and walking was identified for each day using the HSC analysis program and then averaged over the valid days. In addition, distributions of activities were described separately for workplace, working days, and non-working days. Results were reported in % wear time and in h/day.

#### 2.3.3. Participant Characteristics

The questionnaire included age, gender, and education information. Education was defined as low when without and as high when with postsecondary education. In addition, companies provided information on whether participants were working full-time or part-time and whether they had permanent or temporary contracts.

#### 2.3.4. Data Analyses

Participant characteristics and objectively measured sedentary behaviour were described. Furthermore, it was assessed whether sedentary behaviour was dependent on gender, age, education, and company using Χ^2^ and *t*-tests. In addition, we used these statistical analyses to assess differences in people with and without valid objective data on sedentary behaviour.

Frequencies of perceived barriers and facilitators of workplace sedentary behaviour were described. Χ^2^ tests were used to assess whether the barriers and facilitators reported by at least 10% of the sample were different based on gender, age, education, and workplace sedentary behaviour. Education was analysed as high vs. low; age and workplace sedentary behaviour dichotomized into below or above the median. Statistical analyses were performed in IBM SPSS Statistics version 23 (IBM Corp., Armonk, NY, USA).

## 3. Results

### 3.1. Participant Characteristics

In total, 547 office workers consented to participate, and data on perceived barriers and facilitators were available for 533 persons (26%). Of the analytical sample, median age was 41 years (IQR = 35–48), 65% women, 66% highly educated, and the majority had full-time (88%) and permanent (89%) positions. Company A was represented by 39% of participants and company B by 61%.

### 3.2. Sedentary Behaviour

Table 1 shows objectively measured sedentary behaviour, standing, and walking of 311 office workers. Out of 369 participants that consented to wear the inclinometer, 44 did not adhere to the instructions of wearing the inclinometer for at least 4 days and/or filling out the diary, and data of six workers were excluded because >95% of the time was spent in one behaviour. Data of an additional eight participants were excluded because they did not have 2 valid working days. When compared to the total analytical sample, participants with valid inclinometer data were not significantly different in age (*t* = −1.07, *p* = 0.29) and gender (Χ^2^ = 0.54, *p* = 0.47), but more office workers with lower education had valid data (Χ^2^ = 9.95, *p* < 0.01).

Of the analytical sample, almost all had at least 6 valid days (96%), and a vast majority at least 4 working days (82%).

Office workers with high vs. low (median = 68%) workplace sedentary behaviour were not significantly different in age, education, or company, but men were more likely to have high sedentary behaviour compared to women (Χ^2^ = 7.61, *p* < 0.01).

### 3.3. Perceived Barriers

**Figure 1:**
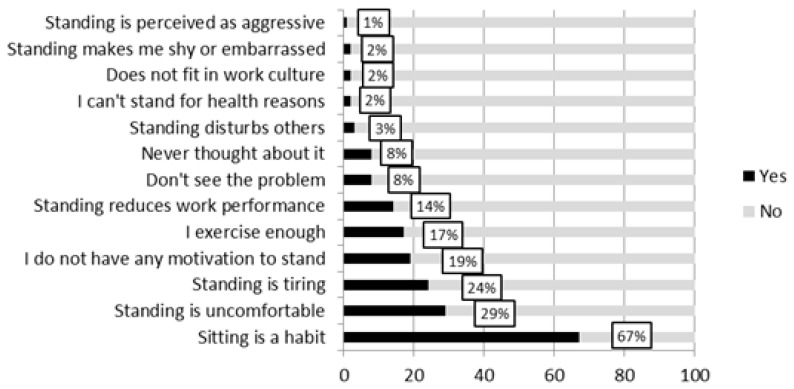
Percentage of office workers reporting proposed barriers for standing up while working, *n* = 533.

Barriers for standing up while working are presented in Figure 1. Sitting is a habit was the most often reported barrier. A higher proportion of younger workers reported that standing is uncomfortable (34% vs. 25% among older office workers, Χ^2^ = 4.57, *p* = 0.04); standing is tiring (30% vs. 16%, Χ^2^ = 14.26, *p* < 0.01), and to have no motivation to stand (23% vs. 15%, Χ^2^ = 5.30, *p* = 0.03). A higher proportion of men reported to exercise enough (22% vs. 13% among women, Χ^2^ = 6.88, *p* = 0.01), whereas a higher proportion of women reported that sitting is a habit (70% vs. 60% among men, Χ^2^ = 5.14, *p* = 0.03). No differences were found comparing office workers with high and low education.

For workplace sedentary behaviour, a higher proportion of participants with high sedentary behaviour reported: sitting is a habit (76% vs. 59% among those with low sedentary behaviour, Χ^2^ = 9.26, *p* < 0.01), standing is uncomfortable (35% vs. 15%, Χ^2^ = 15.12, *p* < 0.01), I do not have any motivation to stand (24% vs. 13%, Χ^2^ = 5.22, *p* = 0.03), and standing reduces work performance (20% vs. 9%, Χ^2^ = 7.43, *p* < 0.01).

### 3.4. Perceived Facilitators

Table 2 presents an overview of proposed facilitators and percentage of office workers that would like the employer to introduce the suggested facilitators. The three most frequently preferred facilitators were the introduction of either standing- or walking-meetings and more possibilities or reminders for breaks. Redesign of own workplace was more frequent among highly educated (14% vs. 4% among lower educated, Χ^2^ = 6.62, *p* = 0.02) and younger workers (10% vs. 5% among older, Χ^2^ = 5.44, *p* = 0.03). No significant differences were found based on gender or workplace sedentary behaviour.

## 4. Discussion

The most frequently reported perceived barrier among office workers was that sitting is a habit. This barrier was more frequent among women than men as well as more frequent among highly sedentary office workers. The two other most reported barriers were that standing is uncomfortable and standing is tiring. These barriers were more frequent among older and among highly sedentary office workers. Facilitators with the most support were the introduction of either standing- or walking-meetings and more possibilities or reminders for breaks.

In contrast to previous qualitative studies reporting cultural and social norms at work as barriers to reduce workplace sitting [8,9,10], this was not found in the present study. However, we were not able to determine whether standing—both at their own workplace and during meetings, presentations, or in other settings—was not perceived as aggressive or made people shy. For a complete understanding of the role of cultural and social norms, future research should look into which barriers apply in different workplace settings. Additionally, further study is needed to assess whether this finding also applies to other cultures, and is not typical for Sweden, as well as to other types of companies.

Our results provide directions for interventions that reduce workplace sedentary behaviour and that show support of office workers: changing standard sitting meetings into either standing- or walking-meetings or to creating more possibilities or reminders for breaks. Surprisingly, a recent Cochrane review reported that no studies were found that investigated the effect of standing- or walking-meetings or of periodic breaks [7]. Therefore, there is a need for randomized, controlled trials studying the effectiveness of these office worker-supported strategies to reduce workplace sedentary behaviour. Nevertheless, even when interventions are successful in decreasing workplace sedentary behaviour, there is an alternative risk that this more favourable behaviour at work will be compensated by increasing sedentary behaviour outside working hours [12,13]. Thus, when focusing on improving sedentary behaviour in one context, overall sedentary behaviour should be taken into account to assure that positive changes in one context did not negatively influence this behaviour in other contexts.

Perceived barriers and facilitators differed based on gender, age, education, and workplace sedentary behaviour. Interestingly, compared to older office workers, younger workers were more likely to report to have no motivation to stand and that standing is tiring or uncomfortable. We did not find that workplace sedentary behaviour was dependent on age, which is in line with a previous study with the same design [14]. However, population-based studies did report associations between younger age and higher self-reported workplace sitting [15,16]. Besides, a higher proportion of men reported to exercise enough. Possibly, this reflects compensatory behaviour, as discussed earlier, as we also found that men were more sedentary compared to women.

Highly educated and younger office workers were more likely to support changes in personal workplace environments. This could potentially partly explain why previous intervention studies targeting the workplace environment reported inconsistent results [7]. Our findings stress the importance of tailored interventions and individualized support in order to reduce sedentary behaviour more effectively.

One previous study in Sweden assessed objectively measured sedentary behaviour among 140 operators, reporting 81% of workplace sitting time. However, this study has several limitations: it was conducted 15 years ago and, during the period after that study, practices probably changed. Further, data were restricted to measurements of less than 6 h with no possibilities to distinguish between standing and walking [17]. Previous studies in Australia and the United Kingdom did use methods comparable to those described in this manuscript and reported higher workplace sedentary times, ranging from 70% to 82% [2,14,18,19], compared to 64% in the current study. While the office workers in the present study were standing as much at the workplace as on non-working days, the office workers in previous studies reported relatively less standing at the workplace compared to outside of work [14,18]. Possibly, this is caused by the availability of sit-stand desks among the office workers in the current study, in distinction to previous studies. However, although sedentary behaviour was slightly lower than in other studies, average workplace sitting time was still higher than what is thought to be desirable for health [1]. Currently, no clear guidelines on sedentary behaviour—in particular, recommended frequency of breaks—are available [1]. Further research is needed to understand the entire scope of physical activity patterns and its relation to health.

Although this quantitative study with objective measurements of sedentary behaviour enhances the currently limited evidence of perceived barriers and facilitators, our findings may not be generalizable to all office workers, companies, or organisations worldwide. Selection bias could have occurred because office workers were included from companies that supported the larger research project, and in particular among those responding. Although the sample size of this study is larger or comparable to previous studies measuring sedentary behaviour with inclinometers, results have to be interpreted with caution because not all participants answering the questionnaire also adhered to the inclinometer measurement. Research in other countries should verify whether barriers and facilitators for sedentary behaviour are the same as among Swedish office workers. Furthermore, experimental and interventional methods are needed to confirm whether manipulation of these barriers and facilitators results in behaviour changes.

## 5. Conclusions

This study provides a detailed description of the ideas that office workers have about sitting and means to reduce sitting. Most office workers perceive that it is difficult to sit less while working because sitting is a habit and that standing is uncomfortable and tiring. Strategies that office workers support include standing- or walking-meetings and creating more possibilities or reminders for breaks. Perceived barriers and facilitators differ based on gender, age, education, and workplace sedentary behaviour. We advise to include the supported strategies as well as to use individualized support in future intervention studies to work towards more effective strategies to reduce workplace sedentary behaviour.

## Figures and Tables

**Table 1:** Description of objectively measured sedentary, standing, and walking time, *n* = 311.

	Workplace	Working Days	Non-Working Days	Overall
Sedentary, %	64 ± 15	61 ± 15	57 ± 17	60 ± 8
h/day	5.5 ± 1.4	10.2 ± 2.5	8.8 ± 2.6	9.7 ± 1.4
Standing, %	27 ± 14	28 ± 10	28 ± 11	28 ± 7
h/day	2.3 ± 1.2	4.6 ± 1.7	4.4 ± 1.7	4.5 ± 1.1
Walking, %	9 ± 3	12 ± 4	14 ± 6	12 ± 3
h/day	0.7 ± 0.2	1.9 ± 0.7	2.1 ± 0.9	2.0 ± 0.5

Percentages are calculated as a proportion of wear time.

**Table 2:** Percentage of office workers reporting proposed facilitators for reducing sedentary behaviour.

	% That Would Like the Employer to Introduce This Facilitator
I don’t see any possibilities	18
Possibility of group support during working hours	7
Individual support from external coach	14
More personal meetings (instead of email etc.)	15
Redesign of work environment	17
Redesign of own workplace	18
Change in attitudes/norms in the workplace	18
App or activity watch	20
Walking meetings	29
Possibilities or reminders for breaks	31
Standing meetings	32

## Supplementary Materials

The following are available online at http://www.mdpi.com/1660-4601/15/4/792/s1, Table S1. Barriers and facilitators to change work-related sedentary behaviour questionnaire in Swedish and English.
