# Supplementary material for: Common Perceived Barriers and Facilitators for Reducing Sedentary Behaviour among Office Workers
Source: Int J Environ Res Public Health. 2018 Apr 18;15(4):792. doi: 10.3390/ijerph15040792 (PMC5923834; doi:10.3390/ijerph15040792)
Supplement: Supplementary file 1 [file ijerph-15-00792-s001.pdf]

Table S1. Barriers and facilitators to change work-related sedentary behaviour questionnaire in Swedish and English.

**Swedish**	**English**	**Answer options**
Anledning till stillasittande<br>*Många faktorer kan orsaka att folk sitter still långa perioder under arbetsdagarna. Vilken/vilka anledningar, om några, på den här listan skulle passa in på dig? Du kan välja flera svarsalternativ*	*Reason for sedentary*<br>*Many factors can cause people to sit for long periods during working days. Which reasons, if any, on this list apply to you? You can choose multiple answer options*	Yes / No
Har aldrig tänkt på det	Never thought about it	
Jag ser inte att stillasittande i långa perioder är ett problem	I don't see prolonged sitting as a problem	
Det är en vana att sitta	Sitting is a habit	
Jag tränar tillräckligt	I exercise enough	
Jag kan inte stå av hälsoskäl	I can't stand for health reasons	
Jag har inte motivation att stå	I do not have any motivation to stand	
Stående är obekvämt	Standing is uncomfortable	
Stående är tröttande	Standing is tiring	
Stående minskar arbetsprestationen	Standing reduces work performance	
Stående stör andra	Standing disturbs others	
Stående uppfattas som aggressivt	Standing is perceived as aggressive	
Jag känner mig blyg eller generad när jag står	Standing makes me shy or embarrassed	
Stående passar sig inte i arbetsplatskulturen	Does not fit in work culture	
Vilket stöd skulle du vilja få från din arbetsgivare för att förändra hur mycket du sitter still.<br>*Du kan välja flera svarsalternativ*	*What support would you like from your employer to change how much you are sitting?*<br>*You can choose multiple answer options*	Yes / No
Utformning av arbetsplatsen som helhet (ex trappor, lunchrum, placering av skrivare)	Redesign of work environment (e.g. stairs, lunch room, placement of printer)	
Utformning av din egen arbetsplats (ex höj- och sänkbart skrivbord, mobiltelefon och datorlösning)	Redesign of own workplace (e.g. standing desks, mobile phone and computer)	
Förändring av attityder/normer på arbetsplatsen	Change attitudes/norms in the workplace	
Stående arbetsmöten	Standing meetings	
Gående arbetsmöten	Walking meetings	
Möjlighet eller påminnelser till pauser el motsvarande	Possibility or reminders for breaks	
Fler personliga möten (istället för e-post, chatt etc.)	More personal meetings (instead of email, chat etc)	
App i mobiltelefon eller aktivitetsarmband	App or activity watch	
Individuellt stöd av extern coach	Individual support from external coach	
Möjlighet till stöd i grupp på arbetstid	Possibility of group support during work hours	
Jag ser inte att min arbetsgivare kan stödja förändring gällande	I don't see any possiblities	
